# The Microbiota-Dependent Worsening Effects of Melatonin on Gut Inflammation

**DOI:** 10.3390/microorganisms11020460

**Published:** 2023-02-11

**Authors:** Jefferson Luiz da Silva, Lia Vezenfard Barbosa, Camila Figueiredo Pinzan, Viviani Nardini, Irislene Simões Brigo, Cássia Aparecida Sebastião, Jefferson Elias-Oliveira, Vânia Brazão, José Clóvis do Prado Júnior, Daniela Carlos, Cristina Ribeiro de Barros Cardoso

**Affiliations:** 1 Department of Clinical Analyses, Toxicology and Food Sciences, School of Pharmaceutical Sciences of Ribeirão Preto, University of São Paulo, Av. do Café, s/n, Ribeirão Preto 14040-903, SP, Brazil; 2 Department of Biochemistry and Immunology, Ribeirão Preto Medical School, University of São Paulo, Ribeirão Preto 14040-903, SP, Brazil

**Keywords:** microbiota, IBD, melatonin, intestinal dysbiosis, inflammation

## Abstract

Dysbiosis and disturbances in gut homeostasis may result in dysregulated responses, which are common in inflammatory bowel diseases (IBD). These conditions may be refractory to the usual treatments and novel therapies are still necessary to reach a more successful regulation of intestinal immunity. The hormone melatonin (MLT) has been raised as a therapeutic alternative because of its known interactions with immune responses and gut microbiota. Hence, we evaluated the effects of MLT in experimental colitis that evolves with intestinal dysbiosis, inflammation and bacterial translocation. C57BL/6 mice were exposed to dextran sulfate sodium and treated with MLT. In acute colitis, the hormone led to increased clinical, systemic and intestinal inflammatory parameters. During remission, continued MLT administration delayed recovery, increased TNF, memory effector lymphocytes and diminished spleen regulatory cells. MLT treatment reduced Bacteroidetes and augmented Actinobacteria and Verrucomicrobia phyla in mice feces. Microbiota depletion resulted in a remarkable reversion of the colitis phenotype after MLT administration, including a counter-regulatory immune response, reduction in TNF and colon macrophages. There was a decrease in Actinobacteria, Firmicutes and, most strikingly, Verrucomicrobia phylum in recovering mice. Finally, these results pointed to a gut-microbiota-dependent effect of MLT in the potentiation of intestinal inflammation.

## 1. Introduction

The intestinal microbiota is essential for numerous physiological processes, including the digestion of food, development of the intestinal immune system and protection against pathogenic microorganisms [1,2,3]. Thus, the dysbiosis and the breakdown of gut homeostasis may result in a dysregulated immune responses and tissue damage [4,5]. Inflammatory bowel diseases (IBD), which include ulcerative colitis and Crohn’s disease, are chronic disorders of the gastrointestinal tract that result from uncontrolled mucosal reactions, related to genetic susceptibility, environmental factors and intestinal microbes [6].

Patients affected by IBD present variations in the bacteria, fungi, viruses and other populations in the intestine, along with an imbalance between commensal and pathogenic microorganisms [7]. As a result of the breakdown in immunological tolerance and gut dysbiosis, inflammatory cells accumulate in the tissues and the exacerbated resulting reactions driven by cytokines such as tumor necrosis factor (TNF), interleukin 1β (IL-1β), IL-17 and IFN-γ [7,8,9] lead to leukocyte activation, epithelial disruption and gut destruction.

IBD treatment requires the use of immunosuppressive and biological drugs, aimed at controlling the intestinal exacerbated inflammation. However, some patients with moderate or severe disease do not respond adequately to the therapeutic interventions and the surgical removal of gut segments are indicated [10]. Thus, novel or adjunctive therapies for IBD would be desirable in an attempt to implement more conservative approaches to regulate intestinal responses. Among a variety of modulatory molecules, hormones such as melatonin (MLT) have been raised as alternatives to modulate intestinal inflammation, because of their known interactions with immune responses and gut microbiota [11,12].

MLT is synthesized predominantly at night by the pineal gland, upon hypothalamic stimulation. Its daily secretion depends on the circadian rhythm synchronized with light/dark cycles, though MLT secretion by extra pineal sources appears to be unrelated to the photoperiod [13]. In the gastrointestinal tract, there are high concentrations of MLT, which may be synthetized by the enterochromaffin cells present in the intestine [14].

Interestingly, the levels of MTL in the intestine are higher than those found in other organs, including the pineal gland, suggesting an important role for this hormone in the gastrointestinal tract [14,15,16,17]. In addition to its effects on immune system cells, recent studies suggest that components of the microbiota such as the intestinal bacteria *Enterobacter aerogenes* are also influenced by the action of MLT [18]. However, despite the apparent relevance of MLT in gastrointestinal immunity, the suitability of this hormone for IBD control is still uncertain [19,20].

Therefore, here, we evaluated the effects of MLT treatment in a mice colitis model that courses with gut dysbiosis, bacteria translocation and exacerbated immune responses, as well as the role of this hormone in the recovery from intestinal inflammation.

## 2. Material and Methods

### 2.1. Animals

Wild-type C57BL/6 male mice, aged 6–8 weeks, were kept in the Animal Facility of the Laboratory of Immunoendocrinology and Regulation (LIR) of the School of Pharmaceutical Sciences of Ribeirão Preto, University of São Paulo (FCFRP/USP), in a clean and silent environment, under normal conditions of humidity and temperature, and with a 12 h light and dark cycle. The mice were given food and water ad libitum throughout the experiment. The procedures were performed in accordance with the principles of ethics in animal research, approved by the Committee on Ethics in the Use of Animals (CEUA) of the FCFRP/USP (Protocol 17.1.1073.60.3).

To evaluate the potential effect of MLT in the colitis model, mice were evaluated in different experimental designs. In the first study protocol, the animals were divided in two groups, comprising those exposed to sodium dextran sulfate (DSS) for 10 days and treated daily with vehicle solution (group 1; n = 5) or MLT (group 2; n = 5), from the 3rd to the 15th experimental day. Under these conditions, the weight and clinical disease scores of the mice were analyzed until the 36th day. Group 3 comprised naive control animals without any experimental intervention (group 3; n = 5). The second study aimed to investigate the influence of MLT in the acute phase of colitis induction. The protocol consisted of mice exposed to DSS for 7 days and treated daily with vehicle solution (group 1; n = 5) or MLT (group 2; n = 5), from the onset of colitis symptoms (3rd day) until the 6th day. Samples were collected on the 7th day. The third protocol was designed to evaluate the effects of MLT in the recovery of intestinal inflammation, after the withdrawal of DSS. Mice were exposed to this colitogenic trigger for 7 days, when the DSS was then removed. The treatment with MLT (group 1; n = 5) or with the vehicle saline (group 2; n = 5) was performed daily, from the 3rd day (symptom onset) until the 12th experimental day. Samples were collected on day 13. The fourth protocol was designed to investigate the role of gut microbiota in the MLT effects on intestinal inflammation. For that, mice received a daily saline solution (group 1, n = 10) or a wide-range antibiotic therapy for microbiota depletion (group 2; n = 10), for 10 days, followed by exposure to DSS for the next 7 days and colitis induction. The control treatment with vehicle solution or MLT was performed from the 3rd day of induction of intestinal inflammation until the 12th day. Similarly, mice were euthanized on day 13, for sample collection and evaluation.

### 2.2. Induction of Experimental Intestinal Inflammation

Colitis was induced with a single cycle of exclusive administration of drinking water containing 3% dextran sulfate sodium (DSS), available to the mice for the period of 7 or 10 days, as described above.

### 2.3. Control and Melatonin Treatments

MLT was administered at a concentration of 10 mg/kg, as previously described [21,22,23], by daily gavage, at 8 a.m. The timing of melatonin administration was chosen based on the initial experiments aimed at characterizing the clinical responses to the treatment as depicted in Supplementary Figure S1. MLT dilution was performed in 0.9% isotonic saline containing 1% ethanol. For the control, mice received the vehicle solution, without MLT, which was also administered by gavage.

### 2.4. Depletion of the Gut Microbiota

To deplete the intestinal microbiota, mice were treated with a combination of five antibiotics diluted in sterile water, as follows: sodium ampicillin (New Farm, Farmaceutical Industry, Anápolis, Goiás, Brazil—1 g/L), neomycin sulfate (Chemical and Farmaceutical Galena LTDA, Campinas, São Paulo, Brazil—1 g/L), metronidazole (Embrafarma Chemical Products and Farmaceutical, São Paulo, Brazil—1 g/L), gentamicin sulfate (Pharmanostra, Rio de Janeiro, Brazil—1 g/L), L) and vancomycin hydrochloride (New Farm, Farmaceutical Industry, Anápolis, Goiás, Brazil—0.5 g/L). The antibiotic therapy was performed by daily gavage, for 10 consecutive days, prior to the DSS and MLT exposure.

### 2.5. Evaluation of Clinical Disease Score

Mice were evaluated daily for weight change and clinical signs of colitis. Each signal (moist perianal region, presence of diarrhea, pus, blood in the stool, hypoactivity and/or piloerection) scored one point and the sum of them, per mouse, corresponded to the daily clinical disease score. Mice that lost more than 5% and less than 10% of their weight in a 24 h period were assigned 1 point and those with a loss superior to 10% received 2 points which were added to the daily score. In the absence of weight reduction or other colitis signs, the score was zero. The total score corresponded to the sum of all points per day, for each mouse, throughout the experimental protocol. In summary, the quantification of the mice score was performed via a visual evaluation of the signs, in each mouse separately, daily, usually by at least two blinded examiners, as previously described by our group [24,25,26].

### 2.6. Euthanasia and Sample Collection

At the end of the experimental protocols, the mice were euthanized. The blood was collected for sera storage, as well as total and specific cell counting by Panotico staining. The colon was divided into fragments for immunological and tissue inflammation assessment, followed by immediate freezing in liquid nitrogen for ELISA, myeloperoxidase (MPO) and N-acetylglucosaminidase (NAG) assays. There was also the collection of the spleen and mesenteric lymph nodes (MLN) for culture, cytokine measurement and cell phenotyping.

### 2.7. Intestinal Permeability Evaluation by FITC-Dextran

The intestinal permeability assay was performed to evaluate the barrier integrity using the FITC-Dextran quantification method. In summary, food and water were withdrawn 12 h before euthanasia, followed by oral administration of the permeability marker at a concentration of 44 mg/100 g body weight (FITC-Dextran, MW 4000; FD4; Sigma-Aldrich, St. Louis, MO, USA). Serum was collected 4 h later and the fluorescence intensity was determined by a fluorimetry reading (excitation, 483 nm; emission, 525 nm). Serum FITC-Dextran concentrations were determined using a standard curve generated by serial dilution of this compound, ranging from 0.06 to 2000 µg/mL.

### 2.8. Total and Differential Leukocyte Counts

Global cell counts were performed in a Neubauer chamber, after diluting whole blood in Turk’s solution. The results were expressed per mL of blood and a slide smear was stained with rapid panotic (Laborclin, Pinhais, PR, Brazil), which uses the Romanowsky hematological staining principle. Cells were quantified using a microscope (DM750, Leica, Wetzlar, Germany) with an oil immersion objective. The examination was performed on the thinnest part of the extension, counting a total of 100 leukocytes (neutrophils, mononuclear cells, eosinophils and basophils).

### 2.9. Indirect Quantification of Neutrophil and Macrophage Activities

The indirect quantification of the presence of neutrophils and macrophages in colonic fragments was performed by determining the activity of the enzymes myeloperoxidase (MPO) and N-acetylglucosaminidase (NAG), respectively. Briefly, intestinal fragments were homogenized and red blood cells were lysed. The remaining cells were lysed for enzyme extraction, which were quantified in the cell extract supernatant. For the determination of MPO, the supernatant was incubated with tetramethylbenzidine (TMB) (BD Bioscience, San Diego, CA, USA) and the absorbance was read at 450 nm. To measure NAG, the same MPO supernatant was used, with the addition of p-nitrophenyl-2-acetamide-β-D-glucopyranoside and citrate buffer. After incubation, the reaction was read at 405 nm. All results were expressed as optical density (OD) corrected for tissue weight in grams (g).

### 2.10. Enzyme Immunoassay for Cytokine Measurement by ELISA

Intestinal fragments were weighed and homogenized in 500 μL of buffer containing protease inhibitor (Complete ^®^—Roche, Pharmaceuticals, Mannheim, Germany). The samples were centrifuged at 5000× *g* for 15 min at 4 °C and the supernatants were used for ELISA reactions (Enzyme-Linked Immunosorbent Assay, BD PharmingenTM, San Diego, CA, USA). The samples were incubated with capture and detection antibodies for IL-17A and TNF, according to the manufacturer’s recommendations (BD BiosciencesTM—San Jose, CA, USA). The reactions were developed with the addition of TMB and stopped with 4M H_2_SO_4_. The readings were performed in a spectrophotometer at 450 nm, and the final concentration was normalized by the weight of the gut fragment used in the assay and was expressed as pg/mL/g tissue.

### 2.11. Immunophenotyping of Spleen Cells and Mesenteric Lymph Nodes

For phenotypic characterization of the T effector memory cells (TEM) and regulatory T cells (Tregs) from the spleen and MLN, we used antibodies against CD3, CD4, CD8, CD62L and CD44. For Tregs, the surface (CD3, CD4, CD25) and intracellular antigens (Foxp3 transcription factor) were labeled. To quantify the production of cytokines by T lymphocytes, cells were stained for the detection of CD3, CD4, IL-4 and IL-10 markers. Antibodies used for labeling were conjugated to the fluorochromes FITC, PE, PeCy-7, BV510, BV421, APC, ALEXA 647 or PERCP (BD PharmingenTM, San Diego, CA, USA), including the respective isotype controls (BD PharmingenTM, San Diego, CA, USA). Cell acquisition was performed in a flow cytometer (LSRFortessa—BD Bioscience^TM^, San Diego, CA, USA) and the analyses were performed using the FlowJo^TM^ v10 software.

For surface immunostaining, cells were divided into specific tubes and incubated with 50 μL of PBS/1% denatured milk for 30 min at 4 °C, to block nonspecific binding sites. Then, incubation was performed with antibodies specific to CD3, CD4, CD8, CD62L or CD44, for 30 min at 4 °C. Subsequently, the cells were washed with PBS, fixed in PBS 1% formalin and later acquired in the flow cytometer.

For Tregs, samples were immunostained, after fixation and permeabilization using the BD PharmingenTM Mouse FoxP3 Buffer Set (BD PharmingenTM, San Diego, CA, USA), according to the manufacturer’s instructions.

To evaluate the production of cytokines (IL-10 and IL-4), leukocytes were restimulated in vitro for 4 h with 50 ng/mL of phorbol-12-myristate-13-acetate (PMA—Sigma-Aldrich, St. Louis, MO, USA) and 500 ng/mL of ionomycin (Sigma-Aldrich, St. Louis, MO, USA), in the presence of 1 μL/mL of brefeldin (Golgi stop, BD BioscienceTM, San Diego, CA, USA) at 37 °C and 5% CO_2_. The leukocytes were stained for surface markers, fixed and permeabilized using the BD PharmingenTM Transcription Factor Buffer Set (BD PharmingenTM, San Diego, CA, USA), followed by incubation with anti-cytokine antibodies for intracellular staining.

### 2.12. Analysis of the Gut Microbiota

To define the relative abundance of bacteria phyla in the gut microbiota, we used feces samples from mice exposed to DSS, treated or not with antibiotics, MLT or vehicle solutions, in quantitative PCR (q-PCR) assays. Briefly, fecal DNA extraction was performed according to the recommendations of the DNeasy^®^ PowerSoil^®^ kit (Qiagen, Hilden, Germany). For PCR analysis, 10 ng of DNA and 1 uM of forward and reverse primers (Eubacteria—normalizer gene16S rRNA primer, Firmicutes, Bacteroidetes, Actinobacteria, Proteobacteria and Verrucomicrobia) were used [27]. The primer sequences are described in Table 1. Differences (ΔCT) between Eubacteria cycle threshold (CT) values and the evaluated phyla were used to obtain normalized levels of each bacteria phylum (2^−ΔΔCT^). The experimental group that received only DSS was used as a normalizer to define the relative abundance of each phylum.

### 2.13. Statistical Analysis

Statistical analyzes were performed using the Graphpad Prism^®^ 6 software (version 6.0). For all variables, normal distribution and homogeneous variance were tested. When the distribution was considered normal and with homogeneous variance, the parametric ANOVA test with Tukey’s post-test was used for three or more groups or Student’s T test for 2 groups. In cases where the distribution was not Gaussian, a nonparametric Kruskal–Wallis ANOVA test was used with Dunn’s post-test in the case of three or more groups or the Mann–Whitney test for 2 groups. Results were expressed as mean ± SEM (standard error of the mean). The differences observed were considered significant when *p* was < 0.05 (5%).

## 3. Results

In the first protocol, to characterize the overall effects of MLT in the experimental intestinal inflammation, we induced colitis in C57BL/6 mice that were treated with this hormone in the acute and repair phases of the disease. The long-term follow-up showed that MLT worsened the DSS-induced intestinal inflammation, especially augmenting the clinical disease score (Supplementary Figure S1). Then, based on these previous findings, we established additional models to evaluate the role of MLT in the modulation of colitis.

**Figure 1:**
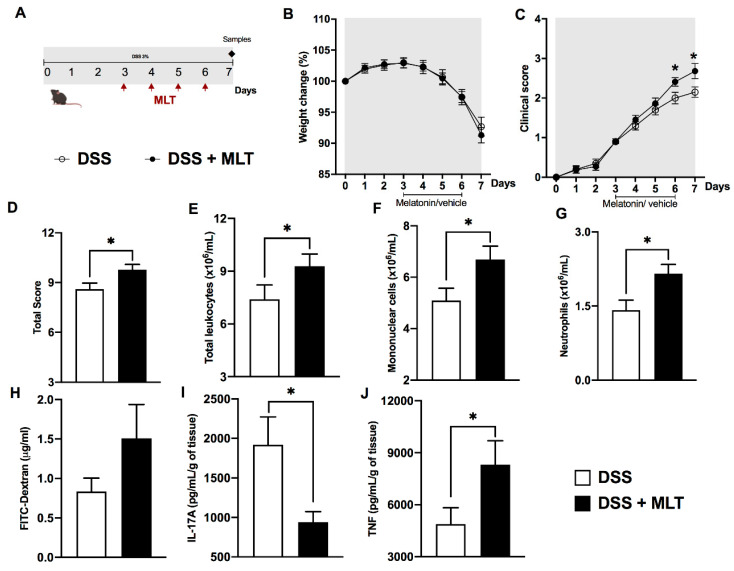
**Melatonin (MLT) potentiates the acute inflammation in experimental colitis.** Intestinal inflammation was induced by exposure to 3% dextran sulfate sodium (DSS) in drinking water for 7 days. (**A**) Mice were treated with MLT (10 mg/Kg) by gavage, from day 3 to 6, and euthanized on day 7 for sample collection (second experimental protocol). In (**B**), changes in body weight related to the first day of colitis induction. (**C**) Daily clinical disease score and (**D**) total score throughout the experimental period. (**E**) Circulating leukocytes, (**F**) mononuclear cells and (**G**) neutrophils. In (**H**), intestinal permeability evaluated by detection of FITC-Dextran in sera samples. (**I**) IL-17A and (**J**) TNF levels in the gut of mice with colitis, treated or not with MLT. The cytokines are depicted in picograms per milliliter, normalized by colon weight. Results are representative of three independent experiments, with five mice/group. * *p* < 0.05.

In the second protocol, mice were exposed to DSS and treated with MLT upon the onset of the inflammation signs (Figure 1). The results revealed a loss of weight and a significant increase in the colitis severity throughout the disease progression, which culminated in a higher total clinical score in mice treated with MLT (Figure 1B–D). In accordance, these animals exhibited augmented numbers of circulating total leukocytes (Figure 1E), including mononuclear cells (Figure 1F) and neutrophils (Figure 1G), thus confirming the systemic inflammatory effects induced by DSS that were potentiated by MLT treatment. Indeed, these mice had a trend towards increased intestinal permeability (Figure 1H), along with significant reduction in IL-17A and a notable TNF production in the inflamed colon (Figure 1I,J).

**Figure 2:**
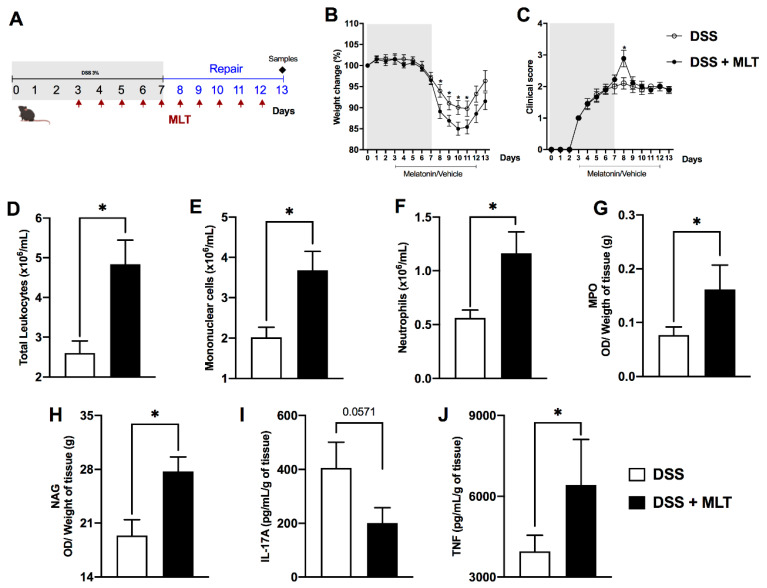
**Melatonin (MLT) impairs the recovery from intestinal inflammation.** Colitis was induced by exposure to 3% dextran sulfate sodium (DSS) in drinking water for 7 days. Mice were treated with MLT (10 mg/Kg) by gavage, daily, from day 3 to 12, and euthanized on day 13 for sample collection ((**A**), third experimental protocol).). In (**B**), changes in body weight related to the first day of colitis induction. (**C**) Daily clinical disease score and (**D**) total score throughout the experimental period. (**E**) Circulating leukocytes, (**F**) mononuclear cells and (**G**) neutrophils. In (**H**), intestinal permeability evaluated by detection of FITC-Dextran in sera samples. (**I**) IL-17A and (**J**) TNF levels in the gut of mice with colitis, treated or not with MLT. The cytokines are depicted in picograms per milliliter, normalized by colon weight. Results are representative of three independent experiments, with five mice/group. * *p* < 0.05.

Next, to verify whether MLT could play a role in the recovery of gut inflammation, the MLT treatment was prolonged until day 13, while the DSS trigger was withdrawn on day 7 (Figure 2A). In this scenario of our third protocol, MLT had a more outstanding influence on the colitis outcome. Hormone-treated mice presented higher weight loss and clinical disease scores than those which did not receive MLT (Figure 2B,C). The worsening of clinical signs was accompanied by elevated numbers of circulating total leukocytes (Figure 2D) such as mononuclear cells (Figure 2E) and neutrophils (Figure 2F). Moreover, the MLT treatment led to an augmented activity of neutrophils and macrophages in the gut mucosa (Figure 2G,H, respectively). Despite not observing statistical differences in IL-17A production (Figure 2I), we noted notable augmented TNF levels in the colon during the remission of intestinal inflammation in the MLT-treated group (Figure 2J).

**Figure 3:**
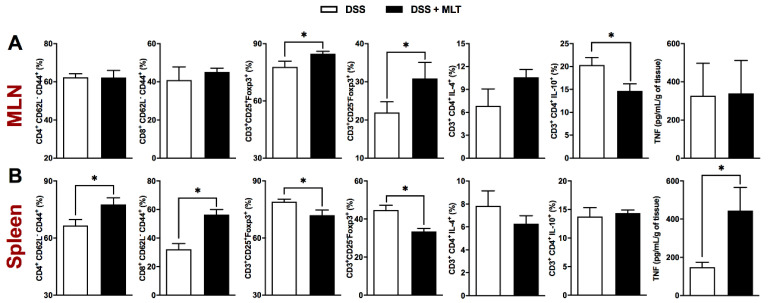
**Spleen and mesenteric lymph nodes (MLN) responses in melatonin (MLT)-treated colitis.** Colitis was induced by exposure to 3% dextran sulfate sodium (DSS) in drinking water for 7 days. Mice were treated with MLT (10 mg/Kg) by gavage, daily, from day 3 to 12, and euthanized on day 13 for sample collection. The frequency (%) of various immune cell subtypes was evaluated by flow cytometry in the MLN (**A**) and spleen samples (**B**). For characterization of effector memory T lymphocytes (TEM), the CD4^+^CD62L^−^CD44^+^ and CD8^+^CD62L^−^CD44^+^ populations were evaluated. The regulatory T cells were characterized by CD3^+^CD25^+^Foxp3^+^ and CD3^+^CD25^-^Foxp3^+^ staining. Identification of CD4 T cells producing IL-4 or IL-10 was also performed in CD3^+^CD4^+^IL-4^+^ and CD3^+^CD4^+^IL-10^+^ populations. The concentration of TNF in both lymphoid organs was determined and expressed in picograms per milliliter, related to the organs’ weight. These results are representative of two independent experiments with five mice per group. * *p* < 0.05.

The regulation of the immune response in the gut-draining lymph nodes was also affected by the MLT treatment of colitis, with an important increase in the frequency of regulatory T cells, despite a reduced CD3^+^CD4^+^ population producing the suppressor cytokine IL-10 (Figure 3A and Supplementary Figure S2). Apart from MLN, the systemic spleen response was notably altered by MLT treatment, with an increased accumulation of TCD4 and TCD8 effector memory cells (Figure 3B and Supplementary Figure S3), in contrast to a diminished frequency of regulatory T cells expressing the Foxp3 regulatory marker (Figure 3B and Supplementary Figure S2). Most interestingly, the splenic TNF production in mice recovering from colitis and treated with MLT was remarkably higher compared to those not treated with this hormone (Figure 3B), suggesting that systemic immune mechanisms related to bacteria control were long-lasting, maintained or potentiated in the face of MLT treatment of intestinal inflammation.

**Figure 4:**
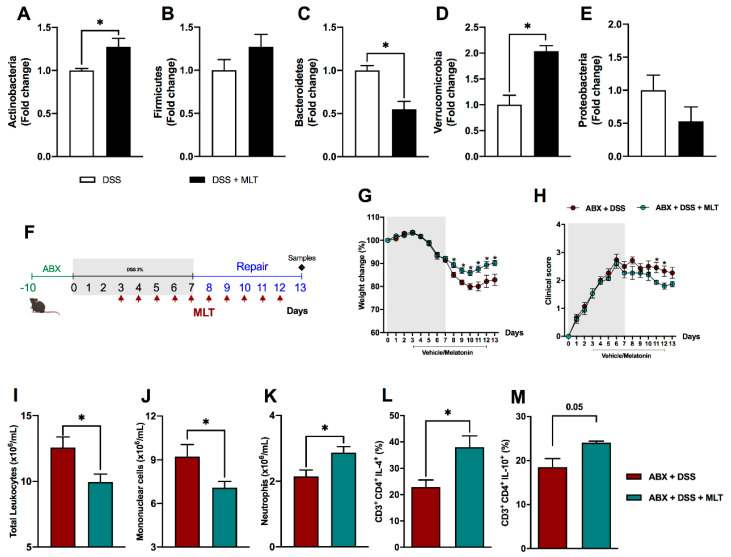
The systemic and persistent inflammation induced by melatonin (MLT) in the recovery phase of experimental colitis is reversed by microbiota depletion. The intestinal inflammation was induced by exposure to 3% dextran sulfate sodium (DSS) in drinking water for 7 days. Mice were treated with MLT (10 mg/Kg) by gavage, daily, from day 3 to 12, and euthanized on day 13 for feces collection. Real-time polymerase chain reaction was performed for detection of Actinobacteria (**A**), Firmicutes (**B**), Bacteroidetes (**C**), Verrucomicrobia (**D**) and Proteobacteria phyla (**E**) in fecal samples. In (**F**), mice were treated daily with antibiotics (ABX) for 10 days for microbiota depletion, prior to colitis induction, as described in Material and Methods (fourth experimental protocol). Afterwards, intestinal inflammation was induced by exposure to drinking water containing 3% sodium dextran sulfate (DSS) for 7 consecutive days. Mice were treated with MLT (10 mg/Kg) by gavage, daily, from day 3 to 12, and euthanized on day 13 for sample collection. (**G**) Weight change (%); (**H**) clinical disease score; (**I**) total circulating leukocytes; (**J**) frequency of peripheral blood mononuclear cells; (**K**) blood neutrophils. In (**L**,**M**), spleen T lymphocytes producing IL-4 or IL-10, respectively. The results are representative of three independent experiments with 5–10 mice per group. * *p* < 0.05.

Subsequently, considering that gut dysbiosis is one of the main features of colitis, we evaluated the impact of MLT on intestinal microbiota composition. Our findings pointed to an outstanding direct or indirect influence of the hormone in the composition of the fecal microbiota, by increasing Actinobacteria in contrast to a reduction in the Bacteroidetes phyla (Figure 4A,C, respectively), while there was no difference in the Firmicutes or Proteobacteria population (Figure 4B,E, respectively). Interestingly, MLT led to a notable increase in Verrucomicrobia phylum in the repair phase of colitis mice treated with this hormone (Figure 4D).

To deeper understand the role of gut dysbiosis in the deterioration of intestinal inflammation and delayed mice recovery upon MLT treatment, animals were subjected to a wide spectrum antibiotic therapy for microbiota depletion, before DSS exposure (Figure 4F), in our fourth experimental protocol. The results showed a remarkable reversion of the disease phenotype with a faster weight recovery and improvement in colitis clinical score in mice that were administered MLT (Figure 4G,H, respectively). The disease amelioration was accompanied by a reduction in blood total leukocytes (Figure 4I), including mononuclear (Figure 4J) and increased neutrophil numbers (Figure 4K). Most importantly, the gut microbiota depletion resulted in a counter regulatory systemic response, as observed with the increase in CD4 T lymphocytes producing IL-4 and IL-10 cytokines in the spleen of mice treated with MLT (Figure 4L,M, respectively).

**Figure 5:**
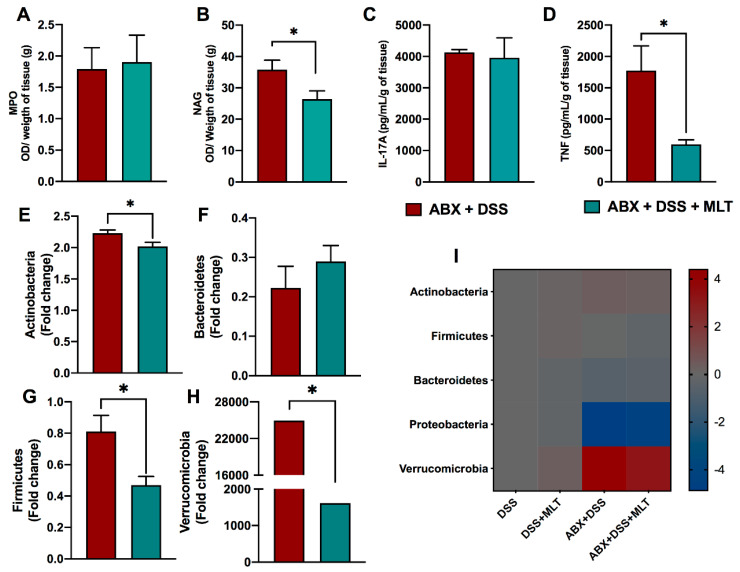
**The melatonin (MLT) effects on longstanding intestinal inflammation and dysbiosis is dependent on gut microbiota.** Mice were treated daily with antibiotics (ABX) for 10 days for microbiota depletion, prior to colitis induction, as described in Material and Methods. Subsequently, intestinal inflammation was induced by exposure to drinking water containing 3% sodium dextran sulfate (DSS) for 7 consecutive days. Mice were treated with MLT (10 mg/Kg) by gavage, daily, from day 3 to 12, and euthanized on day 13 for sample collection. In (**A**,**B**), colon myeloperoxidase (MPO) and N acetylglucosaminidase (NAG) activities. (**C**) IL-17A and (**D**) TNF levels in the gut of mice with microbiota depletion followed by colitis induction and MLT treatment. The cytokines are depicted in picograms per milliliter, normalized by colon weight. Relative expression of (**E**) Actinobacteria, (**F**) Bacteroidetes, (**G**) Firmicutes and (**H**) Verrucomicrobia phyla in fecal samples. (**I**) Heat map summarizing the alterations in the microbiota composition induced by MLT treatment in DSS colitis. The results are representative of three independent experiments with 10 mice per group. * *p* < 0.05.

In line with that, we also observed a reduction in macrophage activity in the gut (Figure 5B) and a marked diminishment in proinflammatory cytokine TNF production in the colon of mice treated with MLT after gut microbiota depletion (Figure 5D). The neutrophil activity and IL-17 responses were not affected in both groups post antibiotic therapy (Figure 5A,C, respectively). Regarding the gut microbiota, the differences in Bacteroidetes were abolished (Figure 5F) and there was not a significant detection of Proteobacteria phylum. Nevertheless, we found a clear reversal of the composition of the other phyla, with a reduction in Actinobacteria (Figure 5E), Firmicutes (Figure 5G) and, most strikingly, Verrucomicrobia phylum (Figure 5H), which was notably increased in the colitis group treated with MLT when gut microbiota was not depleted. Figure 5I represents the heat map summarizing the gut microbiota changes in colitis, which was modulated by the hormonal therapy. Altogether, our data indicates a gut microbiota-dependent effect of MLT in the potentiation of intestinal inflammation induced by DSS.

## 4. Discussion

Disturbances of host microbiota are associated with intestinal disorders that causes with tissue damage and exacerbated immune responses. Alternative approaches aimed at the modulation of mucosal inflammation are fundamental for the control of relapsing chronic diseases that may present with reduced responsiveness to conventional therapies, such as IBD. Hence, since increasing evidence suggests that MLT could play an important role in the relationship between inflammation and gut microbiota [28], we explored its potential modulatory effects in intestinal homeostasis.

MLT is a tryptophan-derived molecule that influences not only circadian rhythm, but also microbial metabolism and leukocyte responses, including the regulation of B and T cells activation [29]. The use of MLT as a possible adjunctive treatment for intestinal diseases has been reported; however, there is no consensus and some studies point to controversial effects on IBD. Furthermore, the exact mechanisms by which the hormone acts on gut immunity are still unclear. Intriguingly, here, we showed that MLT treatment of experimental colitis led to the worsening, instead of the amelioration, of gut inflammation.

The utilization of MLT in experimental models of colitis [30,31,32,33], for a short period of time or at low dosages, was described as beneficial in constraining inflammation. However, in the chronic treatment of TNBS-induced colitis, and in some cases of patients presenting UC or CD, there was a deterioration in intestinal inflammation upon MLT utilization [19,34,35]. Indeed, despite some supposedly beneficial effects described in the literature [36], here, we observed a potentiation of intestinal inflammation in MLT-treated mice, which was dependent on the host gut microbiota.

In our second experimental design, mice were evaluated in the acute phase of inflammation after a four-day MLT administration that was initiated upon the symptom’s onset. Clinical signs, systemic and gut inflammation were accentuated in the hormone-treated group, suggesting a harmful effect on colitis. Differently, in Wistar rats exposed to TNBS, a prophylactic administration of MLT followed by short-term treatment during acute inflammation led to colitis amelioration [35]. On the other hand, the chronic treatment induced the opposite outcome and was in accordance with our findings of a more notable disease worsening in the third experimental design, which included a clear difficulty in weight regaining after DSS withdrawal.

The impaired clinical recovery in both acute and repair phases of colitis in mice treated with MLT could be linked to the increase in circulating leukocyte and TNF production in the colon, despite reduced IL-17 cytokine, which is fundamental for gut immunity in the control of bacteria burden [9]. Corroborating our data, MLT was able to inhibit the differentiation of Th17 cells in an experimental model of necrotizing enterocolitis [37] and, in autoimmune uveitis, MLT suppressed Th17 differentiation through the reactive-oxygen species–TXNIP-HIF1α axis [38]. On the other hand, cytokines with an inflammatory profile intensify mucosal effector responses, though a consequent deterioration in the intestinal lamina propria due to excessive inflammation may occur [39].

The increased number of blood monocytes and neutrophils, besides augmenting myeloperoxidase and macrophages’ activity in the gut of mice exposed to DSS and treated with MLT, is suggestive of inflammation induction and its long-term persistence. Together with the elevated TNF, these findings may point to an important host response to the intestinal dysbiosis and bacteria replication. In fact, macrophages play fundamental functions in the gut, including the resistance to microbiota translocation through the damaged gut barrier and control of intracellular infections. Moreover, circulating blood monocytes are able to increase IL-1β production before migration to the inflamed colon, where these cells are important sources of both IL-1β and TNF [40]. In addition, the neutrophil population, which is also responsible for bacteria elimination, may be markedly increased in the blood and mucosa of IBD patients or in experimental colitis, though its excessive or uncontrolled responses could lead to tissue damage [41,42,43].

The balance between effector, memory and regulatory T lymphocyte populations drive the main cellular responses associated with inflammation and antigen clearance [44,45]. Here, we showed that during disease remission, mice treated with MLT had a higher frequency of effector memory CD4 and CD8 T lymphocytes (TEM) in the spleen, a finding that could be related to reduced regulatory responses by Foxp3^+^ cells in the same lymphoid organ. In accordance, circulating colitogenic CD4 TEM cells have been observed during experimental colitis [46], indicating that sites other than the intestine may present pathogenic lymphocytes able to maintain chronic inflammation. Nevertheless, though the CD3^+^Foxp3^+^ MLN cells were decreased, the concomitant reduction in IL-10 producing lymphocytes at these draining lymph nodes, together with an outstanding splenic TNF production, reiterates the pro-inflammatory potential of MLT in experimental colitis. In line with that, TNF plays a fundamental role in intestinal inflammation as well as in other immune-mediated diseases [47,48].

In addition to the uncontrolled immune response, which can be influenced by genetic and environmental factors, the microbiota is a key element in IBD pathogenesis [49]. These diseases usually present with decreased microbial diversity and gut dysbiosis [50]. *Faecalibacterium prausnitzii* from the Firmicutes phylum and Bacteroidetes are frequently reduced in Crohn’s disease patients, while Proteobacteria and Actinobacteria are commonly increased in comparison to healthy individuals [51,52]. These bacteria translocate through the intestinal epithelium and replicate, causing inflammation [53].

Here, we observed that MLT treatment of colitis-recovering mice led to augmented Actinobacteria in contrast to reduced Bacteroidetes phyla, compared to vehicle treated animals, thus confirming the impairment of gut homeostasis caused by the hormone. Curiously, a consistent augmented expression of the Verrucomicrobia phylum was observed upon MLT treatment, indicating that *Akkermansia muciniphila*, a mucin-degrading bacteria, could be involved in the exacerbation of colitis inflammation in mice with the hormone supplementation [54]. Indeed, the microbiota depletion by a wide-range antibiotic therapy before MLT treatment reversed the main inflammatory and clinical parameters associated with colitis deterioration. These findings were accompanied by a reduction in Actinobacteria, Firmicutes and Verrucomicrobia phyla.

The degradation of mucin by gut bacteria may facilitate IBD onset, due to a facilitation of microbes or antigen access to the gut mucosa, where the local inflammatory response is rapidly triggered. *A. muciniphila* bacteria, a representative of the Verrucomicrobia phylum, worsen the gut inflammatory responses induced by *S. typhimurium* by interfering with local mucus homeostasis [55]. Interestingly, a recent study reported different modulations of gut inflammation dependent on *Akkermansia muciniphila* strains; i.e., while the FSDLZ36M5 isolate protected against colitis, the strains FSDLZ39M14, ATCC BAA-835 and FSDLZ20M4 were not able to induce these beneficial effects. Then, the protective effects assigned to *A. muciniphila* in DSS colitis are strain specific [56]. Indeed, *A. muciniphila* play a dual role in the gut immunity. Despite these bacteria being widely known to constrain the inflammatory response, the opposite effect may occur in mice presenting colorectal cancer, in gnotobiotic animals harboring a specific pathogen or in certain gene deletions, such as in mice not presenting the gene coding for IL-10 [57]. To provide more detail, these bacteria are able to induce spontaneous colitis in germ-free IL10^−/−^ mice, whereas NLRP6 limits their colonization and protects against colitis [57]. Moreover, the replenishment of mice with *A. muciniphila* after antibiotic therapy in colitis associated with colorectal cancer led to damage in the gut barrier, increased bacterial (LPS) translocation as well as augmented local and systemic inflammatory responses [58]. Then, under these conditions, *A. muciniphila* may lead to the worsening of intestinal inflammation [59], thus corroborating our evidence.

As discussed above, our findings are contrary to some previous studies and pointed to an activation effect of MLT on gut immunity, which was dependent on the local microbiota. It is known that mice bred or housed in different facilities may differ in their microbiota and this possibility cannot be ruled out while considering the opposing results presented here. Another innovation of our study is the hormone supplementation only after the initial establishment of the colitis signs, i.e., after the breakdown of mucosal immunity, but before the most severe diseases. In addition, our data are consistent in showing that the immunological parameters corroborated the worsening of gut inflammation in mice treated with MLT, despite the known anti-oxidant effects of this hormone [60]. Interestingly, the mice under the hormonal supplementation not only presented notable signs of colitis aggravation or delayed recovery, but also increased markers of exacerbated inflammatory response. These findings confirmed the deleterious effects of MLT on a previously disrupted gut barrier, indicating that the hormone may have a direct or indirect role in shaping the gut microbiota or the immune response raised to constrain the local dysbiosis. Nevertheless, the amplification of local inflammation may have an undesirable potential to cause persistent tissue damage and intestinal damage.

In conclusion, despite the fact that MLT could play a protective role in specific conditions of inflammation, we still should be cautious regarding its wide use to treat IBD, since hormone supplementation may exacerbate the inflammatory responses, depending on the hosts and on the gut microbiota harbored by them.

## Figures and Tables

**Table 1:** Primers sequences used in real-time PCR.

Primers	Sequences 5′–3′	
Actinobacteria	Sense	TGTAGCGGTGGAATGCGC
	Antisense	AATTAAGCCACATGCTCCGCT
Bacteroidetes	Sense	GTTTAATTCGATGATACGCGAG
	Antisense	TTAASCCGACACCTCACGG
Firmicutes	Sense	ATGTGGTTTAATTCGAAGCA
	Antisense	AGCTGACGACAACCATGCAC
Verrucomicrobia	Sense	TCAKGTCAGTATGGCCCTTAT
	Antisense	CAGTTTTYAGGATTTCCTCCGCC
Eubacteria	Sense	ACTCCTACGGGAGGCAGCAGT
	Antisense	ATTACCGCGGCTGCTGGC

## Data Availability

The data presented in this study are available on request from the corresponding author.

## Supplementary Materials

The following supporting information can be downloaded at: https://www.mdpi.com/article/10.3390/microorganisms11020460/s1, Figure S1: Preliminary characterization of the melatonin (MLT) effects on experimental colitis. Intestinal inflammation was induced by exposure to 3% dextran sulfate sodium (DSS) in drinking water for 10 days. Mice were treated with MLT (10 mg/Kg) by gavage, daily, from day 3 to 15 and followed until day 36 (A, first experimental protocol).). In (B), variation in body weight related to the first day of MLT treatment. (C) Disease clinical score throughout the experimental period. * *p* < 0.05; Figure S2: Gating strategies used to analyze regulatory T cells and intracellular cytokines, stained for flow cytometry acquisition. Lymphocytes were delineated according to size (FSC) and granularity (SSC) parameters, followed by CD3 and CD25 gating to assess the regulatory profile. The intracellular expression of Foxp3 was evaluated in CD3^+^CD25^-^ and CD3^+^CD25^+^ cells by histogram analyzes. CD3^+^ and CD4^+^ T lymphocytes were gated for quantification of the intracellular cytokines IL-10 and IL-4, according to the SSC x cytokine parameters; Figure S3: Gating strategies used to analyze memory T cells, stained for flow cytometry acquisition. Lymphocytes were delineated according to size (FSC) and granularity (SSC) parameters, followed by CD3 gating. For cell subpopulations, the CD4 x CD8 dot plot was used. CD44 expression was then evaluated in the CD62L^+^ and CD62L^-^ population, to define central memory T cells (TCM, CD4^+^CD62L^+^CD44^+^ or CD8^+^CD62L^+^CD44^+^) and effector memory T cells (TEM, CD4^+^CD62L^-^CD44^+^ or CD8^+^CD62L^-^CD44^+^).

## References

[1] De Fazio L., Cavazza E., Spisni E., Strillacci A., Centanni M., Candela M., Praticò C., Campieri M., Ricci C., Valerii M.C. (2014). Longitudinal analysis of inflammation and microbiota dynamics in a model of mild chronic dextran sulfate sodium-induced colitis in mice. World J. Gastroenterol..

[2] Chen Y., Zhou J., Wang L. (2021). Role and Mechanism of Gut Microbiota in Human Disease. Front. Cell. Infect. Microbiol..

[3] Woelk C.H., Snyder A. (2021). Modulating gut microbiota to treat cancer. Science.

[4] Wang Y., Sun L., Chen S., Guo S., Yue T., Hou Q., Feng M., Xu H., Liu Y., Wang P. (2019). The administration of Escherichia coli Nissle 1917 ameliorates irinotecan-induced intestinal barrier dysfunction and gut microbial dysbiosis in mice. Life Sci..

[5] Panpetch W., Hiengrach P., Nilgate S., Tumwasorn S., Somboonna N., Wilantho A., Chatthanathon P., Prueksapanich P., Leelahavanichkul A. (2020). Additional Candida albicans administration enhances the severity of dextran sulfate solution induced colitis mouse model through leaky gut-enhanced systemic inflammation and gut-dysbiosis but attenuated by Lactobacillus rhamnosus L34. Gut Microbes.

[6] Guan Q. (2019). A Comprehensive Review and Update on the Pathogenesis of Inflammatory Bowel Disease. J. Immunol. Res..

[7] Lee J.W.J., Plichta D., Hogstrom L., Borren N.Z., Lau H., Gregory S.M., Tan W., Khalili H., Clish C., Vlamakis H. (2021). Multi-omics reveal microbial determinants impacting responses to biologic therapies in inflammatory bowel disease. Cell Host Microbe.

[8] Abraham C., Cho J.H. (2009). Inflammatory bowel disease. N. Engl. J. Med..

[9] Marié I.J., Brambilla L., Azzouz D., Chen Z., Baracho G.V., Arnett A., Li H.S., Liu W., Cimmino L., Chattopadhyay P. (2021). Tonic interferon restricts pathogenic IL-17-driven inflammatory disease via balancing the microbiome. eLife.

[10] Kassouri L., Amiot A., Kirchgesner J., Tréton X., Allez M., Bouhnik Y., Beaugerie L., Carbonnel F., Meyer A. (2020). The outcome of Crohn’s disease patients refractory to anti-TNF and either vedolizumab or ustekinumab. Dig. Liver Dis..

[11] Zhang B., Chen T., Cao M., Yuan C., Reiter R.J., Zhao Z., Zhao Y., Chen L., Fan W., Wang X. (2022). Gut Microbiota Dysbiosis Induced by Decreasing Endogenous Melatonin Mediates the Pathogenesis of Alzheimer’s Disease and Obesity. Front. Immunol..

[12] Lin R., Wang Z., Cao J., Gao T., Dong Y., Chen Y. (2021). Role of melatonin in murine “restraint stress”-induced dysfunction of colonic microbiota. J. Microbiol..

[13] Soták M., Mrnka L., Pácha J. (2006). Heterogeneous expression of melatonin receptor MT1 mRNA in the rat intestine under control and fasting conditions. J. Pineal Res..

[14] Wang B., Zhu S., Liu Z., Wei H., Zhang L., He M., Pei F., Zhang J., Sun Q., Duan L. (2020). Increased Expression of Colonic Mucosal Melatonin in Patients with Irritable Bowel Syndrome Correlated with Gut Dysbiosis. Genomics Proteomics Bioinform..

[15] Chen C.-Q., Fichna J., Bashashati M., Li Y.-Y., Storr M. (2011). Distribution, function and physiological role of melatonin in the lower gut. World J. Gastroenterol..

[16] Kvetnoy I.M., Ingel I.E., Kvetnaia T.V., Malinovskaya N.K., Rapoport S.I., Raikhlin N.T., Trofimov A.V., Yuzhakov V.V. (2002). Gastrointestinal melatonin: Cellular identification and biological role. Neuroendocrinol. Lett.

[17] Hardeland R., Pandi-Perumal S.R., Cardinali D.P. (2006). Melatonin. Int. J. Biochem. Cell Biol..

[18] Paulose J.K., Cassone V.M. (2016). The melatonin-sensitive circadian clock of the enteric bacterium Enterobacter aerogenes. Gut Microbes.

[19] Calvo J.R., Guerrero J.M., Osuna C., Molinero P., Carrillo-Vico A. (2002). Melatonin triggers Crohn’s disease symptoms. J. Pineal Res..

[20] Trivedi P.P., Jena G.B. (2013). Melatonin reduces ulcerative colitis-associated local and systemic damage in mice: Investigation on possible mechanisms. Dig. Dis. Sci..

[21] Song T.-Y., Lin H.-C., Chen C.-L., Wu J.-H., Liao J.-W., Hu M.-L. (2014). Ergothioneine and melatonin attenuate oxidative stress and protect against learning and memory deficits in C57BL/6J mice treated with D-galactose. Free Radic. Res..

[22] Zhou Q., Lin L., Li H., Wang H., Jiang S., Huang P., Lin Q., Chen X., Deng Y. (2021). Melatonin Reduces Neuroinflammation and Improves Axonal Hypomyelination by Modulating M1/M2 Microglia Polarization via JAK2-STAT3-Telomerase Pathway in Postnatal Rats Exposed to Lipopolysaccharide. Mol. Neurobiol..

[23] Wu H.-M., Zhao C.-C., Xie Q.-M., Xu J., Fei G.-H. (2020). TLR2-Melatonin Feedback Loop Regulates the Activation of NLRP3 Inflammasome in Murine Allergic Airway Inflammation. Front. Immunol..

[24] Sales-Campos H., de Souza P.R., Basso P.J., Ramos A.D., Nardini V., Chica J.E.L., Capurro M.L., Sá-Nunes A., de Barros Cardoso C.R. (2015). Aedes aegypti salivary gland extract ameliorates experimental inflammatory bowel disease. Int. Immunopharmacol..

[25] Basso P.J., Sales-Campos H., Nardini V., Duarte-Silva M., Alves V.B.F., Bonfá G., Rodrigues C.C., Ghirotto B., Chica J.E.L., Nomizo A. (2021). Peroxisome Proliferator-Activated Receptor Alpha Mediates the Beneficial Effects of Atorvastatin in Experimental Colitis. Front. Immunol..

[26] Sales-Campos H., de Souza P.R., Basso P.J., Nardini V., Silva A., Banquieri F., Alves V.B.F., Chica J.E.L., Nomizo A., Cardoso C.R.B. (2017). Amelioration of experimental colitis after short-term therapy with glucocorticoid and its relationship to the induction of different regulatory markers. Immunology.

[27] Leite J.A., Pessenda G., Guerra-Gomes I.C., de Santana A.K.M., André Pereira C., Ribeiro Campos Costa F., Ramos S.G., Simões Zamboni D., Caetano Faria A.M., Candido de Almeida D. (2020). The DNA Sensor AIM2 Protects against Streptozotocin-Induced Type 1 Diabetes by Regulating Intestinal Homeostasis via the IL-18 Pathway. Cells.

[28] Ma N., Zhang J., Reiter R.J., Ma X. (2020). Melatonin mediates mucosal immune cells, microbial metabolism, and rhythm crosstalk: A therapeutic target to reduce intestinal inflammation. Med. Res. Rev..

[29] Luo J., Zhang Z., Sun H., Song J., Chen X., Huang J., Lin X., Zhou R. (2020). Effect of melatonin on T/B cell activation and immune regulation in pinealectomy mice. Life Sci..

[30] Pentney P.T., Bubenik G.A. (1995). Melatonin reduces the severity of dextran-induced colitis in mice. J. Pineal Res..

[31] Liu G., Jiang Q., Chen S., Fang J., Ren W., Yin J., Yao K., Yin Y. (2017). Melatonin alters amino acid metabolism and inflammatory responses in colitis mice. Amino Acids.

[32] Cuzzocrea S., Mazzon E., Serraino I., Lepore V., Terranova M.L., Ciccolo A., Caputi A.P. (2001). Melatonin reduces dinitrobenzene sulfonic acid-induced colitis. J. Pineal Res..

[33] Mazzon E., Esposito E., Crisafulli C., Riccardi L., Muià C., Di Bella P., Meli R., Cuzzocrea S. (2006). Melatonin modulates signal transduction pathways and apoptosis in experimental colitis. J. Pineal Res..

[34] Maldonado M.D., Calvo J.R. (2008). Melatonin usage in ulcerative colitis: A case report. J. Pineal Res..

[35] Marquez E., Sánchez-Fidalgo S., Calvo J.R., la de Lastra C.A., Motilva V. (2006). Acutely administered melatonin is beneficial while chronic melatonin treatment aggravates the evolution of TNBS-induced colitis. J. Pineal Res..

[36] Zhao Z.-X., Yuan X., Cui Y.-Y., Liu J., Shen J., Jin B.-Y., Feng B.-C., Zhai Y.-J., Zheng M.-Q., Kou G.-J. (2021). Melatonin Mitigates Oxazolone-Induced Colitis in Microbiota-Dependent Manner. Front. Immunol..

[37] Ma F., Hao H., Gao X., Cai Y., Zhou J., Liang P., Lv J., He Q., Shi C., Hu D. (2020). Melatonin ameliorates necrotizing enterocolitis by preventing Th17/Treg imbalance through activation of the AMPK/SIRT1 pathway. Theranostics.

[38] Huang J., Li Z., Hu Y., Li Z., Xie Y., Huang H., Chen Q., Chen G., Zhu W., Chen Y. (2022). Melatonin, an endogenous hormone, modulates Th17 cells via the reactive-oxygen species/TXNIP/HIF-1α axis to alleviate autoimmune uveitis. J. Neuroinflammation.

[39] Marafini I., Sedda S., Dinallo V., Monteleone G. (2019). Inflammatory cytokines: From discoveries to therapies in IBD. Expert Opin. Biol. Ther..

[40] Jones G.-R., Bain C.C., Fenton T.M., Kelly A., Brown S.L., Ivens A.C., Travis M.A., Cook P.C., MacDonald A.S. (2018). Dynamics of Colon Monocyte and Macrophage Activation During Colitis. Front. Immunol..

[41] Kühl A.A., Kakirman H., Janotta M., Dreher S., Cremer P., Pawlowski N.N., Loddenkemper C., Heimesaat M.M., Grollich K., Zeitz M. (2007). Aggravation of different types of experimental colitis by depletion or adhesion blockade of neutrophils. Gastroenterology.

[42] Zhong G., Zhang J., Guo Y., Wang Y., Wu M., Ren J., Li Y., Zhang X., Zhou B., Zhao W. (2021). IF1 inactivation attenuates experimental colitis through downregulation of neutrophil infiltration in colon mucosa. Int. Immunopharmacol..

[43] Zhou G., Yu L., Fang L., Yang W., Yu T., Miao Y., Chen M., Wu K., Chen F., Cong Y. (2018). CD177+ neutrophils as functionally activated neutrophils negatively regulate IBD. Gut.

[44] Bishu S., El Zaatari M., Hayashi A., Hou G., Bowers N., Kinnucan J., Manoogian B., Muza-Moons M., Zhang M., Grasberger H. (2019). CD4+ Tissue-resident Memory T Cells Expand and Are a Major Source of Mucosal Tumour Necrosis Factor α in Active Crohn’s Disease. J. Crohn’s Colitis.

[45] Gebhardt T., Whitney P.G., Zaid A., Mackay L.K., Brooks A.G., Heath W.R., Carbone F.R., Mueller S.N. (2011). Different patterns of peripheral migration by memory CD4+ and CD8+ T cells. Nature.

[46] Tomita T., Kanai T., Nemoto Y., Fujii T., Nozaki K., Okamoto R., Tsuchiya K., Nakamura T., Sakamoto N., Totsuka T. (2008). Colitogenic CD4+ effector-memory T cells actively recirculate in chronic colitic mice. Inflamm. Bowel Dis..

[47] Alam M.S., Otsuka S., Wong N., Abbasi A., Gaida M.M., Fan Y., Meerzaman D., Ashwell J.D. (2021). TNF plays a crucial role in inflammation by signaling via T cell TNFR2. Proc. Natl. Acad. Sci. USA.

[48] Schreiber S., Ben-Horin S., Leszczyszyn J., Dudkowiak R., Lahat A., Gawdis-Wojnarska B., Pukitis A., Horynski M., Farkas K., Kierkus J. (2021). Randomized Controlled Trial: Subcutaneous vs Intravenous Infliximab CT-P13 Maintenance in Inflammatory Bowel Disease. Gastroenterology.

[49] Qin J., Li R., Raes J., Arumugam M., Burgdorf K.S., Manichanh C., Nielsen T., Pons N., Levenez F., Yamada T. (2010). A human gut microbial gene catalogue established by metagenomic sequencing. Nature.

[50] Lee M., Chang E.B. (2021). Inflammatory Bowel Diseases (IBD) and the Microbiome-Searching the Crime Scene for Clues. Gastroenterology.

[51] Xu X., Ocansey D.K.W., Hang S., Wang B., Amoah S., Yi C., Zhang X., Liu L., Mao F. (2022). The gut metagenomics and metabolomics signature in patients with inflammatory bowel disease. Gut Pathog..

[52] Ni J., Wu G.D., Albenberg L., Tomov V.T. (2017). Gut microbiota and IBD: Causation or correlation?. Nat. Rev. Gastroenterol. Hepatol..

[53] Vrakas S., Mountzouris K.C., Michalopoulos G., Karamanolis G., Papatheodoridis G., Tzathas C., Gazouli M. (2017). Intestinal Bacteria Composition and Translocation of Bacteria in Inflammatory Bowel Disease. PLoS ONE.

[54] Crouch L.I., Liberato M.V., Urbanowicz P.A., Baslé A., Lamb C.A., Stewart C.J., Cooke K., Doona M., Needham S., Brady R.R. (2020). Prominent members of the human gut microbiota express endo-acting O-glycanases to initiate mucin breakdown. Nat. Commun..

[55] Ganesh B.P., Klopfleisch R., Loh G., Blaut M. (2013). Commensal Akkermansia muciniphila exacerbates gut inflammation in Salmonella Typhimurium-infected gnotobiotic mice. PLoS ONE.

[56] Liu Q., Lu W., Tian F., Zhao J., Zhang H., Hong K., Yu L. (2021). Akkermansia muciniphila Exerts Strain-Specific Effects on DSS-Induced Ulcerative Colitis in Mice. Front. Cell. Infect. Microbiol..

[57] Seregin S.S., Golovchenko N., Schaf B., Chen J., Pudlo N.A., Mitchell J., Baxter N.T., Zhao L., Schloss P.D., Martens E.C. (2017). NLRP6 Protects Il10-/- Mice from Colitis by Limiting Colonization of Akkermansia muciniphila. Cell Rep..

[58] Wang K., Wu W., Wang Q., Yang L., Bian X., Jiang X., Lv L., Yan R., Xia J., Han S. (2022). The negative effect of Akkermansia muciniphila-mediated post-antibiotic reconstitution of the gut microbiota on the development of colitis-associated colorectal cancer in mice. Front. Microbiol..

[59] Luo Y., Lan C., Li H., Ouyang Q., Kong F., Wu A., Ren Z., Tian G., Cai J., Yu B. (2022). Rational consideration of Akkermansia muciniphila targeting intestinal health: Advantages and challenges. NPJ Biofilms Microbiomes.

[60] Reiter R.J., Mayo J.C., Tan D.-X., Sainz R.M., Alatorre-Jimenez M., Qin L. (2016). Melatonin as an antioxidant: Under promises but over delivers. J. Pineal Res..

