# Supplementary material for: The Microbiota-Dependent Worsening Effects of Melatonin on Gut Inflammation
Source: Microorganisms. 2023 Feb 11;11(2):460. doi: 10.3390/microorganisms11020460 (PMC9962441; doi:10.3390/microorganisms11020460)
Supplement: Supplementary file 1 [file microorganisms-11-00460-s001.zip › microorganisms-2146269-supplementary.pdf]

Supplementary figures

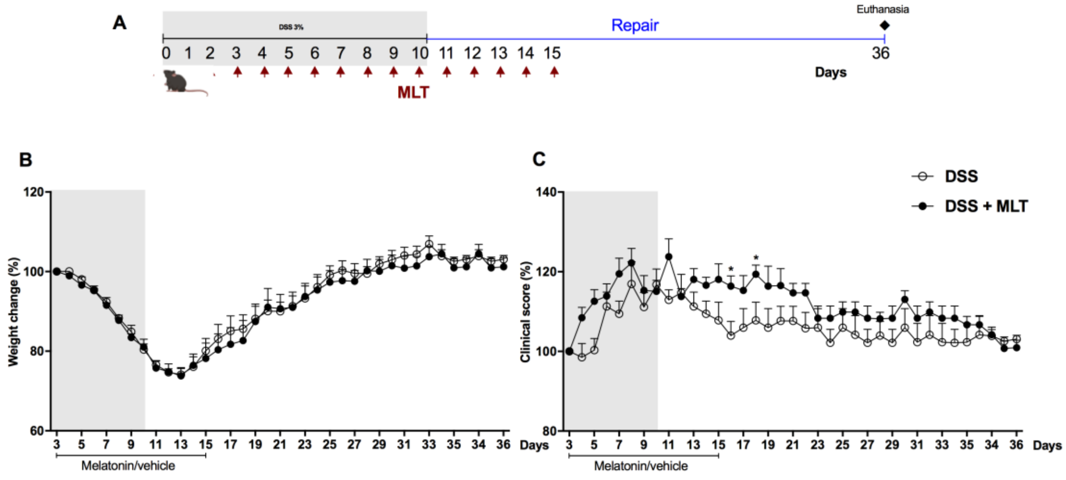

Supplementary Figure S1. Preliminary characterization of the melatonin (MLT) effects on experimental colitis. Intestinal inflammation was induced by exposure to 3% dextran sulfate sodium (DSS) in drinking water for 10 days. Mice were treated with MLT (10 mg/Kg) by gavage, daily, from day 3 to 15 and followed until day 36 (A, first experimental protocol).). In (B), variation in body weight related to the first day of MLT treatment. (C) Disease clinical score throughout the experimental period. * $p < 0.05$**.**

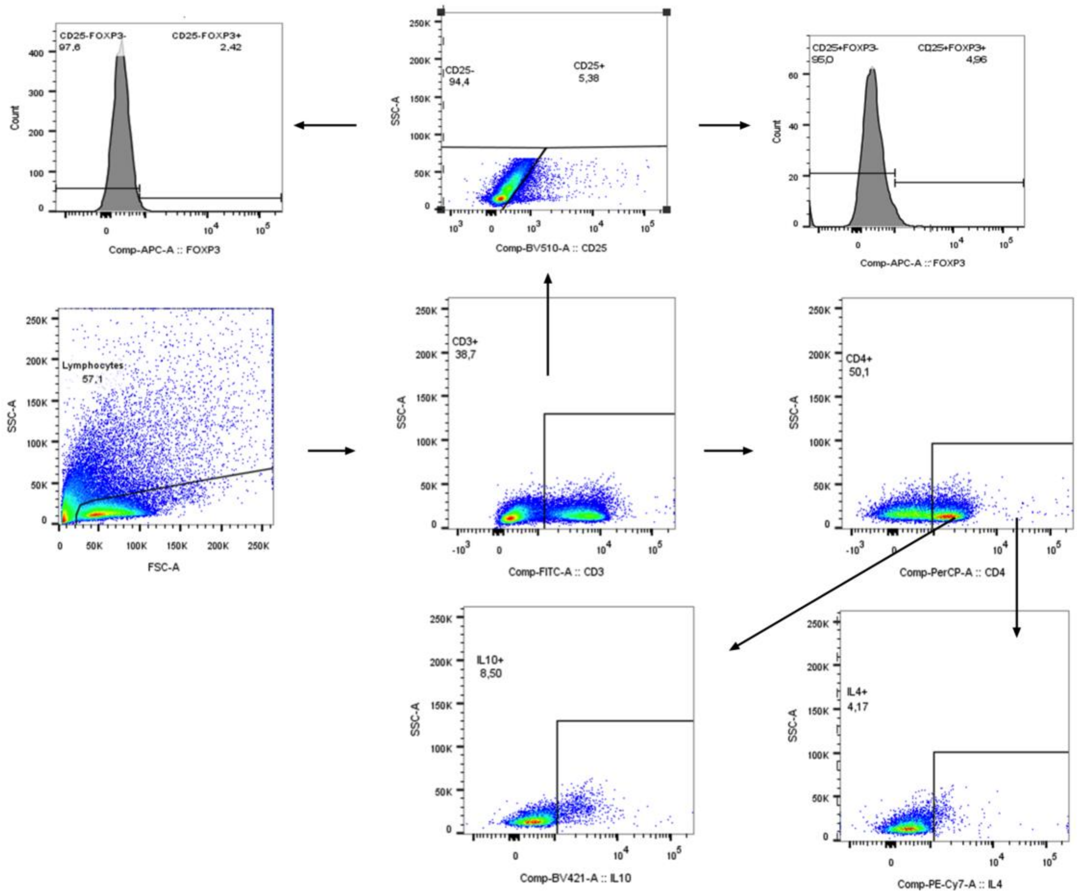

Supplementary Figure S2. Gating strategies used to analyze regulatory T cells and intracellular cytokines, stained for flow cytometry acquisition. Lymphocytes were delineated according to size (FSC) and granularity (SSC) parameters, followed by CD3 and CD25 gating to assess the regulatory profile. The intracellular expression of Foxp3 was evaluated in $CD3^+CD25^-$ and $CD3^+CD25^+$ cells by histogram analyzes. $CD3^+$ and $CD4^+$ T lymphocytes were gated for quantification of the intracellular cytokines IL-10 and IL-4, according to the SSC x cytokine parameters.

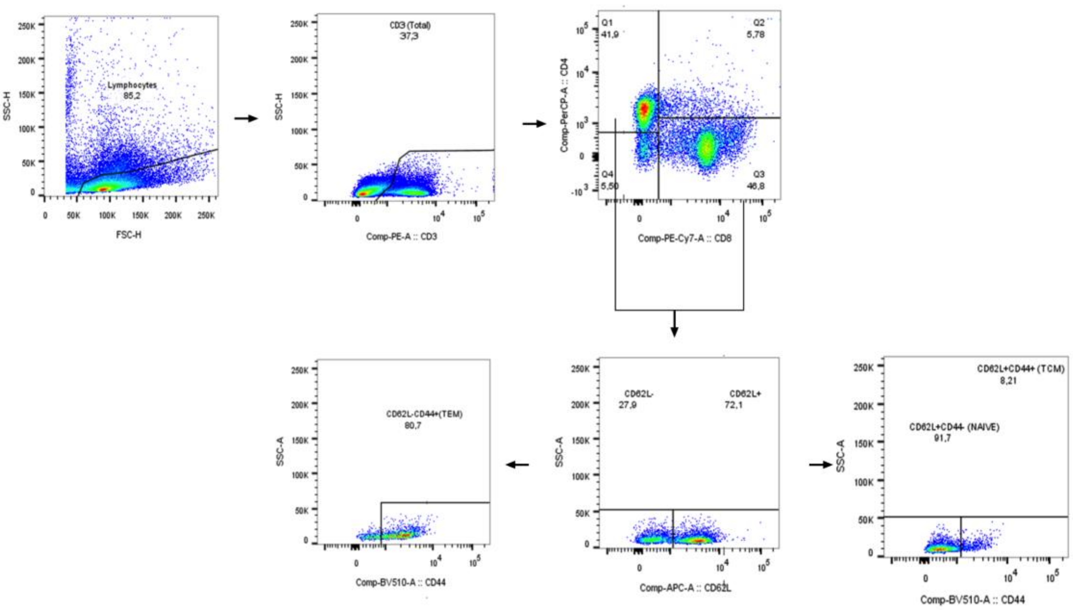

Supplementary Figure S3. Gating strategies used to analyze memory T cells, stained for flow cytometry acquisition. Lymphocytes were delineated according to size (FSC) and granularity (SSC) parameters, followed by CD3 gating. For cell subpopulations, the CD4 x CD8 dot plot was used. CD44 expression was then evaluated in the $CD62L^{+}$ and $CD62L^{-}$ population, to define central memory T cells (TCM, $CD4^{+}CD62L^{+}CD44^{+}$ or $CD8^{+}CD62L^{+}CD44^{+}$) and effector memory T cells (TEM, $CD4^{+}CD62L^{-}CD44^{+}$ or $CD8^{+}CD62L^{-}CD44^{+}$).
